# Preoperative screening for illicit drug use in patients undergoing emergency surgery: A prospective observational study

**DOI:** 10.1038/s41598-018-25829-3

**Published:** 2018-05-09

**Authors:** Jingyi Li, He Ma, Ren Liao, Yanjuan Huang, Guiyong Chen

**Affiliations:** 1 Department of Dermatovenereology, West China Hospital of Sichuan University, Chengdu, 610041 China; 2 Department of Anesthesiology, Nanning Second People’s Hospital, Nanning, 530031 China; 3 Department of Anesthesiology, West China Hospital of Sichuan University, Chengdu, 610041 China; 4 Department of Anesthesiology, Nanning Second People’s Hospital, Nanning, 530031 China; 5 Beijing Biosino-Agiaccu Biotechnology Co. Ltd., Beijing, 102200 China

## Abstract

Knowledge of illicit drug users (IDUs) is important because of the comorbidity related to drug use. In this prospective, observational study, we screened 1007 patients undergoing emergency surgery and found that 75 of them (7.5%) were IDUs The results of preoperative screening showed that the rates of HIV and syphilis infection were significantly higher in IDUs (HIV (+) 2.6%, syphilis (+) 10.7%) than in non-IDUs (HIV (+) 0, syphilis (+) 0.5%). Intraoperative consumption of remifentanil (IDUs: 1.85 ± 1.30 vs. non-IDUs: 1.31 ± 0.86, p = 0.009), midazolam (IDUs: 4.82 ± 1.52 vs. non-IDUs: 4.15 ± 1.81, p = 0.002), and atracurium (IDUs: 31.5 ± 15.1 vs. non-IDUs: 25.5 ± 11.9, p = 0.006) and the proportion of patients requiring postoperative fentanyl (IDUs: 15 (20.0%) vs. non-IDUs: 95 (1.2%), p = 0.031) were significantly increased in IDUs compared to non-IDUs. Postoperative complications were observed in 22.7% (17/75) of patients who were IDUs, which was significantly increased when compared with non-IDUs (6.0%, 56/932, p < 0.001). The mortality rate within 30 days after surgery was similar between the two groups. These findings suggested that the IDUs were associated with increased rates of HIV and syphilis infection; greater consumption of intraoperative opioids, sedatives, and muscle relaxants; increased postoperative complications and a similar mortality rate within 30 days after surgery when compared with non-IDUs.

## Introduction

Illicit drug use is defined as the use of illegal drugs such as heroin, methamphetamine, lysergic acid diethylamide, and marijuana; aberrant non-medical use of pharmaceutical drugs including prescription opioids such as pethidine, morphine, and fentanyl; the use of sedatives such as diazepam; or the use of anesthetics such as ketamine. According to the UNODC (United Nations Office on Drugs and Crime) 2012 World Drug Report from the WHO (World Health Organization), approximately 230 million people, 1 out of every 20 people, took illicit drugs at least once in 2010^1^. The Ministry of Public Security registry alone documented a total of 2.955 million drug abusers in 2014 in China^2^. Given the estimation techniques, the actual population of illicit drug users (IDUs) could be five times the registered number^1^; thus, the anticipated number of IDUs may be more than 14 million in China^2^. The extensive use of illicit drugs has led to an increase in adverse events including traffic accidents caused by the influence of drugs on driving, trauma from fighting after drug abuse, or delivery of a baby by parturient IDUs, and increases in the incidence of drug-induced disability and death have been reported in recent decades^3^.

Treatment of IDUs in the perioperative period, especially for emergency surgery, is challenging. It is important for the anesthesiologists and surgeons to be aware of which drugs are used by the patient and the frequency of their preoperative use; however, the limited knowledge of a patient’s history of drug use due to impaired communication or concealment of drug abuse may lead to insufficient preoperative preparation^3^. Illicit drugs or administration techniques may result in life-threatening complications^4^ (e.g., pseudoaneurysm at the injection site and limb ischemia) to further complicate the situation. Perioperative management may be complicated by medical or psychiatric co-morbidities; psychological states presenting as delirium, hallucination, or difficult behavior; drug effects related to intoxication, withdrawal, tolerance, or opioid-induced hyperalgesia^5^; and the patient’s social disruption or attitudes toward treatment^6^. Additionally, awareness of the drug’s pharmacodynamics, pharmacokinetics, and interactions with anesthetics and other medications is crucial to the proper management of IDUs during the perioperative period^7^. Moreover, it is not uncommon for IDUs to have infection with human immunodeficiency virus (HIV), hepatic C virus (HCV), syphilis, or other sexually transmitted diseases (STD)^8–10^, which may put medical staff in danger, especially those in the window period, in which the infection can not be detected but may still infect others.

It is important to understand the situations of IDUs, such as the category of drugs used, the dosage, the duration of drug use, their history of detoxification or abstention, and especially their current abuse of drugs. With little information about the history of drug abuse, a quick screening for recent use of drugs is important for both medical staff and patients. The immune colloidal gold (ICG) technique is a well established technique and is commonly used by the Ministry of Public Security of the People’s Republic of China for screening for drug abuse, especially for suspicion of driving while under the influence of drugs^2^. The application of the ICG test cassette is simple and easy to learn; a small sample of saliva, urine, or blood is placed into the test cassette, and it then takes only a few minutes to obtain a positive or negative result for the current use of illicit drugs including cannabis, cocaine, opioids (e.g., morphine, pethidine, and tramadol), ketamine, amphetamine, and midazolam.

The aim of this prospective observational study was to identify the IDUs in patients undergoing emergency surgery by applying the ICG test cassette for preoperative screening, in order to help physicians to get a better knowledge of IDU in clinical practice.

## Results

A total of 1007 patients undergoing emergency surgery were screened from May 27, 2016 to January 25, 2017, and none of the patients declined to participate in this study. No patient presented with a history of illicit drug use, but 75 patients (7.45%) were found to be positive for illicit drug use. Of the IDUs, 50 (4.97%) tested positive for morphine, 22 (2.18%) tested positive for methamphetamine, 13 (1.29%) tested positive for ketamine, 6 (0.60%) tested positive for two drugs, and 2 (0.20%) tested positive for all three drugs.

There were no differences in the male to female ratio, age, height, weight, or occupation between IDUs and non-IDUs. In terms of level of education, 24.0% of the IDUs and 11.7% of the non-IDUs had a high school-level education, and none of the IDUs had a college-level education. For the blood test, the red blood cell count, leukocyte count, platelet count, serum hemoglobin concentration, albumin, urea nitrogen, and glucose levels were comparable between the IDUs and non-IDUs. The results of preoperative screening for HBV and HCV were not different between the two groups, and the numbers of HIV-positive and syphilis-positive results were significantly higher in the IDUs (HIV (+) 2.6%, syphilis (+) 10.7%) than in the non-IDUs (HIV (+) 0%, syphilis (+) 0.5%) (Table 1).

**Table 1:** Characteristics of patients.

		IDUs	Non-IDUs	Total	P-value
Number		75 (7.47%)	932 (92.53%)	1007	
Male/Female (No.)		50/25	648/284	698/309	0.345
Age (years)		41.0 ± 5.9	42.9 ± 17.8		0.353
Height (cm)		163.0 ± 9.8	163.1 ± 8.5		0.883
Weight (kg)		60.3 ± 13.3	59.8 ± 11.8		0.728
Occupation — no. (%)	Unemployed	56 (74.4%)	580 (62.2%)	636 (63.2%)	0.479
	Manual workers	5 (6.7%)	97 (10.4%)	102 (10.1%)	
	Peasant	5 (6.7%)	103 (11.1%)	108 (10.7%)	
	Clerk	1 (1.3%)	25 (2.7%)	26 (2.6%)	
	Freelance	0	10 (1.1%)	10 (1.0%)	
	Retired	4 (5.3%)	36 (3.9%)	40 (4.0%)	
	Student	4 (5.3%)	81 (8.7%)	85 (8.4%)	
Degree of education — no. (%)	Illiterate	8 (10.7%)	89 (9.5%)	97 (9.6%)	**0**.**012**
	Primary school	20 (26.7%)	238 (25.5%)	258 (25.6%)	
	Middle school	29 (38.7%)	463 (49.7%)	492 (48.9%)	
	High school	18 (24.0%)	109 (11.7%)	127 (12.6%)	
	College	0	33 (3.5%)	33 (3.3%)	
Comorbidity — no. (%)	None	55 (74.3%)	722 (78.4%)	777 (78.1%)	0.109
	Hypertension	5 (6.8%)	70 (7.6%)	75 (7.5%)	
	Coronary disease	1 (1.4%)	6 (0.7%)	7 (0.7%)	
	Diabetes	2 (2.7%)	31 (3.4%)	33 (3.3%)	
	Hyperthyroidism	0	2 (0.2%)	2 (0.2%)	
	Cerebral infarction/hemorrhage	2 (2.7%)	27 (2.9%)	29 (2.9%)	
	Pulmonary infection/tuberculosis	0	14 (1.4%)	14 (1.4%)	
	Gastritis/gastric ulcer	6 (8.1%)	33 (3.6%)	39 (3.9%)	
	Renal dysfunction	1 (1.4%)	7 (0.8%)	8 (0.8%)	
	Gout	1 (1.4%)	5 (0.5%)	6 (0.6%)	
Red blood cell count (No. ×10^12^)		4.50 ± 0.76	4.37 ± 0.57		0.141
Hemoglobin level (g/L)		126.9 ± 16.8	124.0 ± 16.5		0.149
Platelet count (No. ×10^9^)		248.4 ± 74.7	255.3 ± 65.2		0.387
Leukocyte count (No. ×10^9^)		10.8 ± 4.5	10.6 ± 3.0		0.624
Albumin (g/L)		38.2 ± 5.6	38.1 ± 3.5		0.964
Urea nitrogen		4.6 ± 2.1	4.9 ± 1.2		0.277
Blood glucose		5.89 ± 1.72	5.78 ± 1.15		0.629
Hepatitis B (+) (No. %)		17 (22.7%)	244 (26.2%)	261 (25.9%)	0.303
Hepatitis C (+) (No. %)		1 (1.3%)	3 (0.3%)	4 (0.4%)	0.267
HIV (+) (No. %)		**2 (2**.**6%)**	**0**	**2 (0**.**2%)**	**0**.**005**
Syphilis (+) (No. %)		**8 (10**.**7%)**	**5 (0**.**5%)**	**13 (1**.**3%)**	**<0**.**001**

The anesthetic time and surgical time were comparable between the two groups. The intraoperative consumption of remifentanil, midazolam, and atracurium and the proportion of patients requiring postoperative fentanyl were significantly increased among the IDUs compared to the non-IDUs. 22.7% (17/75) of patients in the IDU group had postoperative complications, compared to 56/932 (6.0%) in the non-IDU group (p < 0.001). Although the preoperative co-morbidity of pulmonary infection/tuberculosis was comparable between the two groups (IDUs: 0 vs. non-IDUs: 1.4%), the incidence of postoperative pulmonary infection was significantly higher in the IDUs (14.7%, 11/75) compared with that in the non-IDUs (5.0%, 47/932). There was no significant difference in mortality rate within 30 days after surgery between the two groups (Table 2).

**Table 2:** In-hospital anesthetic medication consumption, postoperative complications and mortality rate.

		IDUs	Non-IDUs	Total	P value
Number		75 (7.47%)	932 (92.53%)	1007	
Anesthetic time (min)		214.3 ± 131.6	192.2 ± 96.2		0.159
Surgical time (min)		151.7 ± 120.5	139.6 ± 84.9		0.397
Intra- operative anesthetic medication consumption	Fentanyl (mg)	0.26 ± 0.14	0.24 ± 0.12		0.350
	Remifentanil (mg)	**1**.**85 ± 1**.**30**	**1**.**31 ± 0**.**86**		**0**.**009**
	Midazolam (mg)	**4**.**82 ± 1**.**52**	**4**.**15 ± 1**.**81**		**0**.**002**
	Propofol (mg)	972.0 ± 684.3	797.0 ± 501.4		0.070
	Atracurium (mg)	**31**.**5 ± 15**.**1**	**25**.**5 ± 11**.**9**		**0**.**006**
Patients who required fentanyl in the postoperative period — no. (%)		**15 (20**.**0%)**	**95 (1**.**2%)**	**110 (10**.**9%)**	**0**.**031**
Postoperative complication — no. (%)	None	**58 (77**.**3%)**	**876 (94**.**0%)**	**934 (92**.**8%)**	**<0**.**001**
	Pulmonary infection	**11 (14**.**7%)**	**47 (5**.**0%)**	**58 (5**.**8%)**	
	Arrhythmia	**2 (2**.**7%)**	**0**	**2 (0**.**2%)**	
	Deep venous thrombosis	**0**	**2 (0**.**2%)**	**2 (0**.**2%)**	
	Coagulation disorder	**3 (4**.**0%)**	**1 (0**.**1%)**	**4 (0**.**4%)**	
	Renal dysfunction	**1 (1**.**3%)**	**2 (0**.**2%)**	**3 (0**.**3%)**	
	Nerve injury	**0**	**4 (0**.**4%)**	**4 (0**.**4%)**	
Mortality rate within 30 days after surgery — no. (%)		3 (4.0%)	17 (1.8%)	20 (2.0%)	0.182

## Discussion

In this prospective observational study screening for preoperative illicit drug use involving 1007 patients undergoing emergency surgery, we found that 7.45% (75/1007) of the patients had recently used illicit drugs, which was much higher than the 0.2% (2.955 million/1.4 billion) of registered drug abusers in China according to the Chinese official report in 2014^2^. Even considering that the actual number may be five times the number of IDUs registered using the estimating technique, the proportion of 7.45% found in this study would still much higher than the proportion of 1% for the general population. However, of all the patients who screened positive for drugs, none presented with a history of illicit drug use. With the application of the Morphine & Methamphetamine & Ketamine Diagnostic Kit (Beijing Biosino-Agiaccu Biotechnology Co. Ltd.) for screening for recent illicit drug use (detectable time period from 6 hours to 2 weeks after drug use), the test results could be observed in 5 minutes. For the IDUs determined by screening, special care should be taken during the perioperative period.

During surgery, the consumption of remifentanil, midazolam, and atracurium was increased in the IDUs compared to that in the non-IDUs. More patients in the IDU group required postoperative fentanyl for analgesia compared with the non-IDU group. Fentanyl and remifentanil are strong opioid receptor agonists, and the opioid receptors were down-regulated with chronic opioid addiction^11^, resulting in tolerance and dependence. Midazolam is a weak agonist at the delta and kappa opioid receptors^12^, and it exacerbates opioid tolerance^13^. Perhaps that is the reason the IDUs needed more intraoperative remifentanil and midazolam and postoperative fentanyl. For the IDUs with opioid tolerance, the increase in atracurium should be explored in future studies.

Consistent with previous reports^9,10,14^, high rates of HIV and syphilis infection were observed in the IDUs. IDUs have an increased risk for infection with microbial pathogens such as HIV, HCV, and Treponema pallidum because of their exposure to risk factors including shared syringes, contaminated drug paraphernalia, and unprotected sexual activity after drug use^10,14,15^. In consideration of both the management of patients and protection of medical staff from nosocomial infections, preoperative drug screening should be performed in patients undergoing emergency surgery. Although none of the IDUs experienced drug overdoses or abstinence syndrome, incidences of postoperative complications were significantly increased in these patients compared with non-IDUs, especially pulmonary infections and coagulation disorders. Based on these findings, it is suggested that intense care for infection and coagulation should be applied after surgery for IDUs.

To the best of our knowledge, this is the first report concerning preoperative screening for illicit drug use in patients undergoing emergency surgery. The results could provide information about drug use in the surgical population. There were some limitations in this study. First, by application of the ICG test cassette, a drug user could be identified within a few minutes, and the current use of certain drugs such as cannabis, cocaine, opioids, ketamine, or amphetamines could be determined at the same time. However, information concerning the length of time the patient had been taking the illicit drugs could not be ascertained. Second, false-positive results could be found in some patients who recently took cough syrup or prescription drugs for the common cold that contained ephedrine or pseudoephedrine. Third, the sample size was relatively small; thus, a large multicenter trial needs to be conducted in the future.

In conclusion, the proportion of illicit drug users was higher among patients undergoing emergency surgery than in the general population. The rates of HIV infection and syphilis were increased in IDUs. More opioids, midazolam, and muscle relaxants should be used after the identification of an IDU to achieve satisfactory anesthesia. The incidences of postoperative complications, especially pulmonary infections and coagulation disorders, were increased in the IDUs, while the mortality rate was the same as that for the non-IDUs.

## Methods

### Study design and participants

This study was a prospective, observational cohort study that preoperatively screened for IDUs before emergency surgery. The study was conducted at West China Hospital in Chengdu and Nanning Second People’s Hospital in Nanning, China.

**Figure 1:**
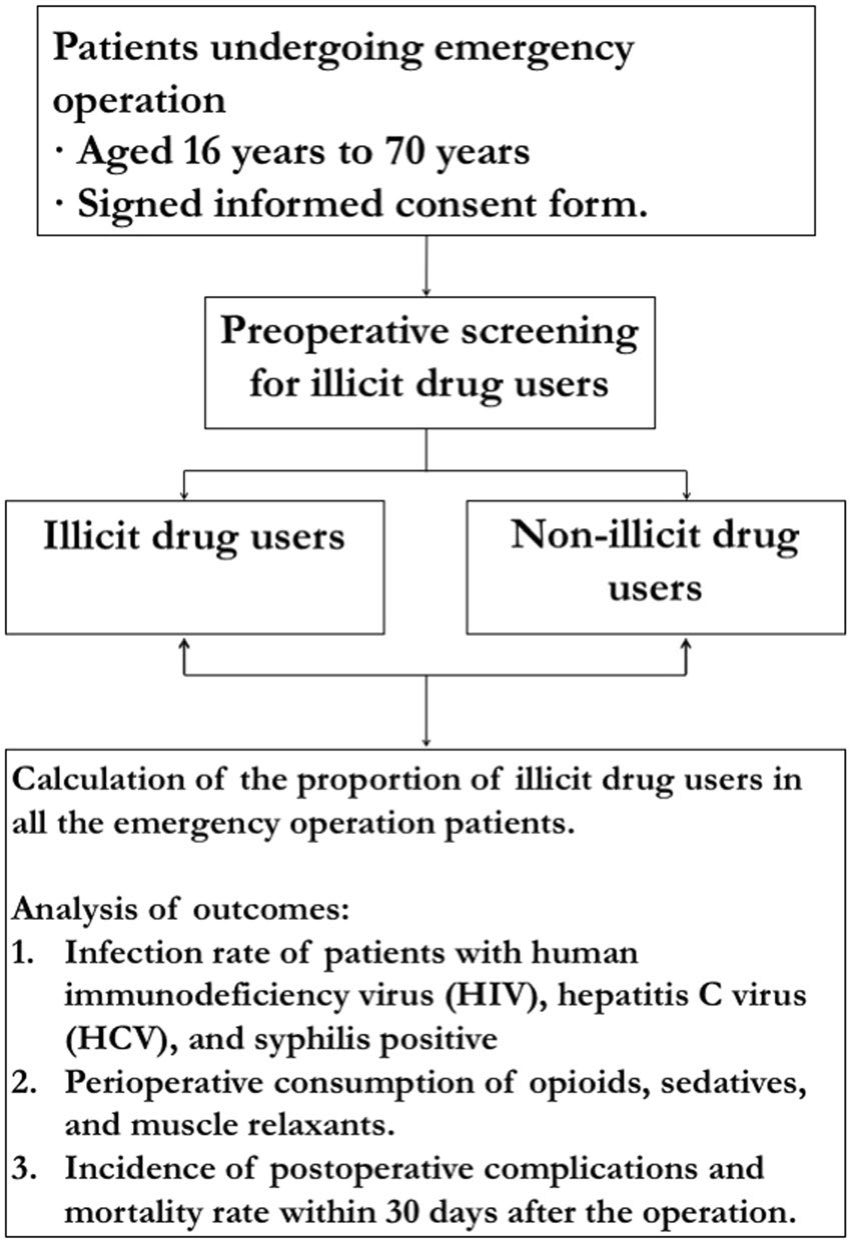
The study algorithm.

The protocol of the study has been registered at http://www.clinicaltrials.gov (NCT 02621411), and a brief flowchart of the study is summarized in Fig. 1. This study was conducted according to the principles outlined in the Declaration of Helsinki. The funding source was the West China scientific research project (H1509073) with grant support from Beijing Biosino-Agiaccu Biotechnology Co. Ltd., who provided the Morphine & Methamphetamine & Ketamine Diagnostic Kit for preoperative screening. Other than financial sponsorship, the company had no role in the study protocol development, implementation, or data collection and analysis. The authors and their colleagues were responsible for the trial design and execution, related statistical analyses, and all aspects of manuscript preparation, including drafting, editing, and decisions on the final content. All investigators in participating centers were appropriately qualified by training to conduct the trial. All patients had to sign the informed consent form prior to study entry.

### Ethics

This study protocol was approved by the Biological- Medical Ethical Committee of West China Hospital of Sichuan University, Chengdu, Sichuan, China on June 1^st^, 2015 and then approved by Nanning Second People’s Hospital review boards. The investigators thoroughly explained the details of the study to the potential subjects and/or the legal guardian, and a signed informed consent form was provided by all eligible patients before enrollment. Participation in the study was entirely voluntary, and patients could withdraw from the study at any time. The privacy of all participants was protected. Personal medical records could be viewed only by investigators and inspectors, and they would not export any confidential information. Data anonymity was applied throughout the entire process of data management, and the data of all subjects were centrally held.

### Procedures

This study began on May 27, 2016, and follow-up was completed on January 25, 2017; the number of patients was determined by time during this period. Patients 16–70 years old who were undergoing emergency surgery were enrolled for illicit drug screening. We excluded patients who enrolled in another research study or had been taking experimental medication during the 3 months prior to this study. Additionally, patients with conditions that may have precluded them from the study, such as a language barrier or psychiatric disorders, were not enrolled. After arrival at the operating room, the patient was catheterized, and the Morphine & Methamphetamine & Ketamine Diagnostic Kit (colloidal gold, BioSino-AgiAccu, China) was applied for preoperative screening for illicit drug use. Two or three drops of urine (approximately 100 µl) were extracted from the indwelling urethra catheter and slowly squeezed into the specimen area of the kit. The test result was observed in the detection time window of the 3rd to 5th minute from the moment that the urine drops were applied to the specimen area.

#### Sensitivity of the Morphine & Methamphetamine & Ketamine Diagnostic Kit

This kit is used to qualitatively detect morphine in human saliva at a minimum detectable concentration of 15 ng/ml, methamphetamine at a minimum detectable concentration of 50 ng/ml, and ketamine at a minimum detectable concentration of 100 ng/ml.

#### Specificity of the Morphine & Methamphetamine & Ketamine Diagnostic Kit

Morphine: Results were positive if the cross-reacting material revealed normorphine in concentrations greater than or equal to 100 µg/ml; ranitidine and procaine in concentrations greater than or equal to 20 µg/ml; or codeine, pholcodine, heroin and O6-monoacetylmorphine in concentrations greater than or equal to 15 ng/ml.

Methamphetamine: The results were positive if the cross-reacting material revealed an ephedrine concentration greater than or equal to 100 ng/ml; a phenylephrine hydrochloride concentration greater than or equal to 15 g/ml; and ranitidine and dextral methamphetamine concentrations greater than or equal to 20 g/ml.

Ketamine: The results were positive if the cross-reactive substances revealed methadone, at 150 µg/ml and methamphetamine at 150 µg/ml.

**Figure 2:**
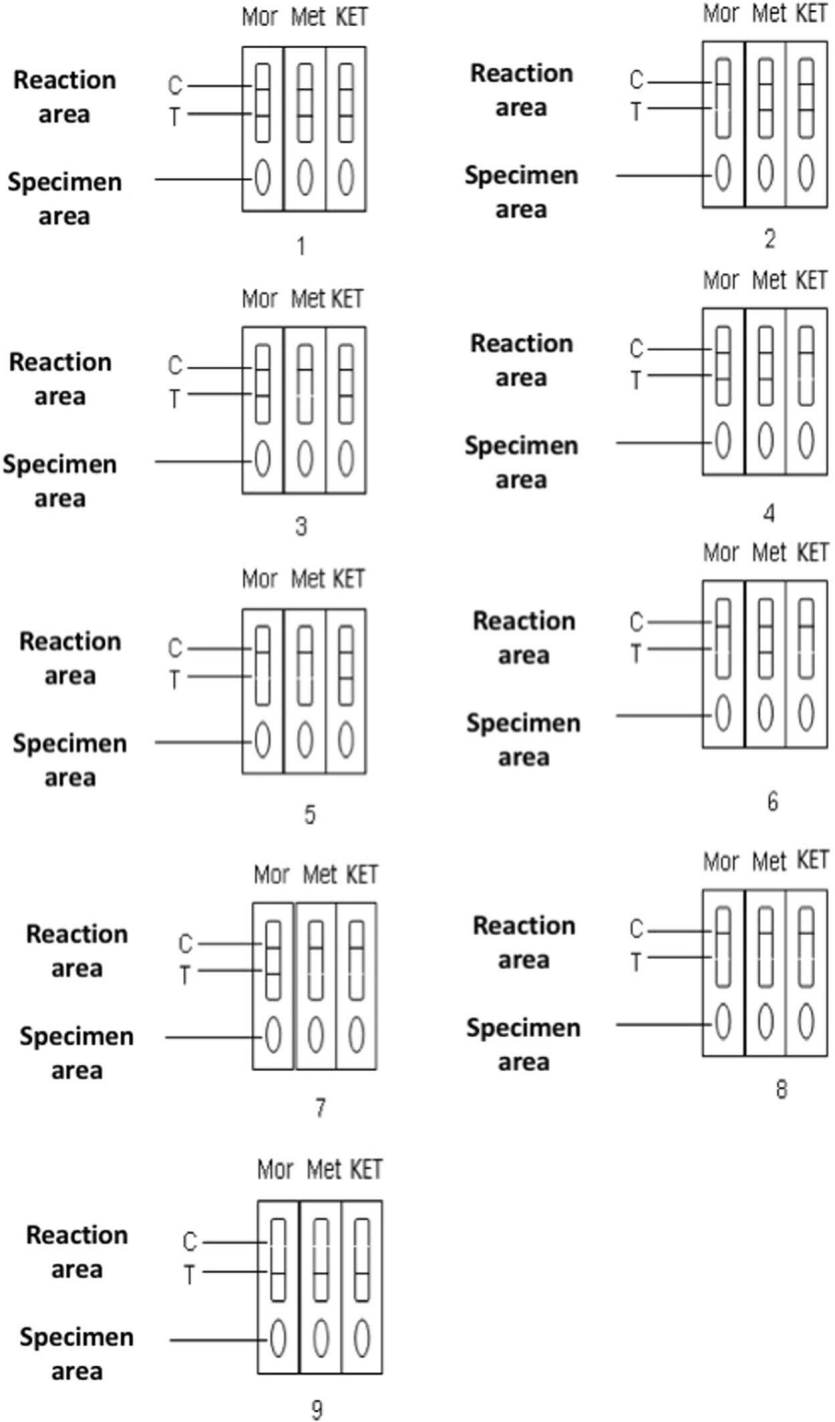
Interpretation of the Morphine & Methamphetamine & Ketamine Diagnostic Kit. (1) Positivity: There is only one red control line (C) in the reaction area. (2) Negativity: There are a red control line (C) and test lines (T) in the reaction area. (3) Invalid: There is no control line (C) in the reaction area, no matter whether there is a test line (T) or not; in this case, retesting with a new cassette device is required.

The interpretation of the results is shown in Fig. 2 and Table 3.

**Table 3:** Interpretation of the Morphine & Methamphetamine & Ketamine Diagnostic Kit.

Results No.	C	Mor	Met	KET	Result
1	appear	appear	appear	appear	Mor/Met/KET all negative (−)
2	appear	disappear	appear	appear	Met/KET both negative (−), Mor positive (+)
3	appear	appear	disappear	appear	Mor/KET both negative (−), Met positive (+)
4	appear	appear	appear	disappear	Mor/Met both negative (−), KET positive (+)
5	appear	disappear	disappear	appear	Mor/Met both positive (+), KET negative (−)
6	appear	disappear	appear	disappear	Mor/KET both positive (+), Met negative (−)
7	appear	appear	disappear	disappear	Met/KET both positive (+), Mor negative (−)
8	appear	disappear	disappear	disappear	Mor/Met/KET all positive (+)
9	disappear	Despite whether Mor/Met/KET appear	Invalid repeat with a new cassette		

All the eligible patients were followed up from the time of the illicit drug screening to discharge. After the completion of data collection, the proportion of IDUs was calculated as the number of IDUs divided by the total number of enrolled patients, and patients were divided into the IDU group and the non-IDU group. The infection rate of patients with serum HIV, hepatitis B, hepatitis C, or syphilis; the intraoperative consumption of fentanyl, remifentanil, midazolam, propofol, and atracurium; the incidence of postoperative complications; and the mortality rate within 30 days after surgery were compared between the two groups.

### Statistical analysis

The data analysis was performed using SPSS (Statistical Product and Service Solutions) 18.0 software (SPSS Inc., Chicago, Illinois, USA). Data were analyzed for distribution (by Kolmogorov-Smirnov goodness-of-fit test) and homogeneity (C-variances test) and expressed as the means ± standard deviation (for normal distribution) or medians (for skewed distribution). Quantitative data were compared using analysis of variance (ANOVA), and qualitative data were analyzed using the χ^2^ test (chi-square test). A P-value < 0.05 was considered significant.

### Data availability statement

All the materials, data and associated protocols can be made available to readers upon request.
